# Role of umbilicocerebral and cerebroplacental ratios in prediction of perinatal outcome in FGR pregnancies

**DOI:** 10.1007/s00404-021-06268-4

**Published:** 2021-10-02

**Authors:** H. Coenen, J. Braun, H. Köster, M. Möllers, R. Schmitz, J. Steinhard, K. Oelmeier

**Affiliations:** 1 Department of Gynecology and Obstetrics, University Hospital Münster, Albert-Schweitzer-Campus 1, 48149 Münster, Germany; 2 Center for Prenatal Medicine and Human Genetics, Münster, Germany

**Keywords:** Cerebroplacental ratio, Umbilicocerebral ratio, Fetal Growth Restriction, Perinatal outcome, Doppler Ultrasound

## Abstract

**Purpose:**

Aim of our study was to compare the prognostic value of the Umbilical-to-Cerebral ratio (UCR) directly to the Cerebroplacental ratio (CPR) in the prediction of poor perinatal outcomes in pregnancies complicated by Fetal Growth Restriction (FGR).

**Methods:**

A retrospective study was carried out on pregnant women with either a small-for-gestational age (SGA) fetus or that were diagnosed with FGR. Doppler measurements of the two subgroups were assessed and the correlation between CPR, UCR and relevant outcome parameters was evaluated by performing linear regression analysis, binary logistic analysis and receiver operator characteristic (ROC) curves. Outcomes of interest were mode of delivery, acidosis, preterm delivery, gestational age at birth as well as birthweight and centiles.

**Results:**

Boxplots and Scatterplots illustrated the different distribution of CPR and UCR leading to deviant correlational relationships with adverse outcome parameters. In almost all parameters examined, UCR showed a higher independent association with preterm delivery (OR: 5.85, CI 2.23–15.34), APGAR score < 7 (OR: 3.52; CI 1.58–7.85) as well as weight under 10th centile (OR: 2.04; CI 0.97–4.28) in binary logistic regression compared to CPR which was only associated with preterm delivery (OR: 0.38; CI 0.22–0.66) and APGAR score < 7 (OR: 0.27; CI 0.06–1.13). When combined with different ultrasound parameters in order to differentiate between SGA and FGR during pregnancy, odds ratios for UCR were highly significant compared to odds ratios for CPR (OR: 0.065, 0.168–0.901; *p* = 0.027; OR: 0.810, 0.369–1.781; *p* = 0.601). ROC curves plotted for CPR and UCR showed almost identical moderate prediction performance.

**Conclusion:**

Since UCR is a better discriminator of Doppler values in abnormal range it presents a viable option to Doppler parameters and ratios that are used in clinical practice. UCR and CPR showed equal prognostic accuracy conserning sensitivity and specificity for adverse perinatal outcome, while adding UA PI and GA_scan increased prognostic accuracy regarding negative outcomes.

**Supplementary Information:**

The online version contains supplementary material available at 10.1007/s00404-021-06268-4.

## Introduction

Fetal Growth Restriction (FGR) is a serious obstetric complication affecting 5–10% of pregnancies worldwide [1]. It is associated with an increased risk of adverse perinatal outcome, such as premature birth, fetal hypoxia, neonatal acidosis, low APGAR score or intrauterine death [2, 3]. There are multiple causes for FGR—they can be of fetal, placental or maternal origin such as preeclampsia. Ultimately, they lead to the same endpoint: insufficient uteroplacental perfusion and restricted fetal nutrition which is reflected by abnormal Doppler parameters [4].

Currently, there is controversy regarding the definition of FGR. This condition is most commonly defined as the fetus failing to reach its genetically predetermined growth potential.

In this context, fetuses with an estimated weight below the 10th centile are referred to as “small for gestational age “(SGA). Sometimes, the terms “FGR” and “SGA” are even used synonymously. This has led to uncertainty regarding the diagnosis of FGR. In the current guideline on the diagnosis and management of FGR, the ISUOG (International Society of Ultrasound in Obstetrics and Gynecology) states that the fetal size alone is not sufficient to identify FGR, unless abdominal circumference (AC) or estimated fetal weight (EFW) is below the 3rd centile. To distinguish between SGA and FGR Doppler velocimetry of uteroplacental and fetoplacental circulations may be used [5]*.*

The current German guidelines define SGA and FGR as follows: SGA pregnancies show an estimated fetal weight below the 10th centile without further detectable pathologies such as abnormal Doppler of the umbilical artery or oligohydramnios. In contrast, FGR is defined as EFW below the 10th centile and/or a drop in fetal growth velocity combined with a resistance in the pulsatility index (PI) of the umbilical artery (UA) or uterine artery PI above the 95th centile and/or the presence of oligohydramnios [6].

According to the ISUOG guidelines, two phenotypes of FGR are distinguished: by definition early onset FGR is diagnosed before 32 weeks of gestation with alterations of fetal circulation being determined by placental insufficiencies leading to high morbidity and mortality rates. In contrast, late onset FGR (> 31 + 6 weeks of gestation) is based on more unspecific placental lesions and a reduced tolerance to hypoxemia in fetuses near term, correlating with poor perinatal outcomes [5].

In the absence of effective treatment options, the major challenges in FGR pregnancies are the assessment of intrauterine fetal risks and optimal timing of delivery. The surveillance of pregnancies affected by FGR has improved through advances in Doppler ultrasonography. Due to different underlying pathomechanisms of early- and late-onset FGR that result in specific pregnancy surveillance and management strategies, different Doppler parameters are useful in their detection and monitoring.

In early-onset FGR, reduced placental perfusion is reflected by an increased UA PI, mean cerebral artery (MCA), as well as an increased ductus venosus (DV) pulsatility.

Especially in cases with late-onset FGR, it has been shown that as a response to long-term hypoxia, the perfusion of the brain increases with a reduction of vascular resistance in the MCA PI, also described as the “brain-sparing effect”.

Quantified as the cerebroplacental ratio (CPR), the ratio MCA PI/UA PI is said to reflect alterations in placental or fetal blood flow more sensitively than the UA PI or MCA PI alone [7, 8]. In cases where the presence of an FGR is questionable (e.g., presence of oligohydramnios but Doppler parameters within normal range), using a ratio that includes two different areas of blood flow can provide additional diagnostic insight and can be an indicator for a manifested FGR.

To estimate the optimal timing of delivery, it is essential to use prediction parameters with high sensitivity. However, recent literature indicates variable accuracy for predicting adverse outcomes with CPR, making its clinical utility controversial [9–11]. Latest publications suggest that the umbilicocerebral ratio (UCR), which is the inversion of the CPR, is a more sensitive predictor for various adverse perinatal outcome parameters [12–14]. Despite being reversed ratios calculated from the same Doppler values, the TRUFFLE study reported better correlations of the UCR with neonatal neurodevelopmental impairment [12]. A different study demonstrated a correlation between UCR and low umbilical cord pH and a strong association with an adverse composite outcome in pregnancies affected by gestational diabetes, whilst CPR did not show any correlation [14].

To the best of our knowledge, most publications report the predictive value of the CPR rather than the UCR and so far studies directly comparing the predictive potential of poor perinatal outcomes in FGR pregnancies are rare.

The aim of our study was to compare UCR with CPR and other established ultrasound parameters in their prediction of negative outcomes in pregnancies complicated by FGR and SGA fetuses.

## Materials and methods

In our single-center study, we retrospectively evaluated SGA and FGR pregnancies presenting for routine ultrasound examinations at the author′s department. Ultrasound examinations were carried out by specialists in prenatal diagnosis and perinatal care. The study was designed according to the Declaration of Helsinki and was approved by our Institutional Review Board.

Gestational age (GA) was calculated using the crown-rump length during the first trimester of pregnancy. SGA and FGR were defined as described in the current German guideline on Fetal Growth Restriction [6]. Doppler measurements were performed according to the standard recommendations of ISUOG practice guidelines: in absence of fetal breathing movements the MCA was visualized at the level of the sphenoid bones close to its origin at an insonation angle below 30°. The sample volume was placed in the center of the vessel and blood flow parameters were measured after obtaining at least three similar consecutive waveforms. The UA PI was assessed in a free-floating loop using a corresponding technique [15]. As previously described, CPR was calculated as the ratio between MCA PI and UA PI. Accordingly, we calculated the UCR as the ratio of the UA PI and MCA PI [18].

Data acquisition was carried out between 2005 and 2019. Only singleton SGA and FGR pregnancies between 24 and 40 weeks of gestation with complete follow-up were included in this study. Exclusion criteria were maternal age below 18 years, major fetal malformation or aneuploidy, as well as fetal infection. If more than one Doppler measurement was recorded, the one closest to delivery was chosen to maximize prognostic accuracy.

Images were acquired using an iU22 and EPIQ7 (Philips Medical Systems, Andover, MA, USA) and Toshiba Aplio ultrasound systems (Toshiba Medical Systems, Tokyo, Japan). Doppler measurements, fetal estimated weight as well as data on pregnancy outcome were obtained from our hospital data base (ViewPoint, GE Healthcare, Fairfield, CT, USA).

Relevant outcome parameters were mode of delivery, umbilical cord pH, APGAR score at five minutes, GA at birth (GA_birth), birth weight and birth weight centiles. Adverse outcome was defined as umbilical cord pH < 7.21, 5-min APGAR score < 7, premature birth < 37th week, extremely premature birth < 30th week, obstetric intervention (operative delivery or cesarean section [CS]) or low (< 10th centile) or very low birth weight (< 3rd centile).

### Statistical analysis

Before running tests that assume a normal distribution of the data, we ran log transformations to reduce skewness. The Kolmogorov–Smirnov and Shapiro–Wilk test were used to assess the distribution of the data, boxplots and histograms were used to visualize the distributions of CPR and UCR. Binary logistic regression analysis and multivariate logistic regression analysis were performed to assess the association between UCR and CPR and the different outcome parameters birth weight, GA, umbilical cord pH, GA_birth, mode of delivery, birth weight centiles, and APGAR score as well as determining FGR or SGA. Multiple ultrasound parameters were combined to detect predictive markers with a maximized sensitivity. Scatterplots, boxplots and histograms were generated to graphically visualize and compare the different test models of CPR and UCR.

Receiver operating characteristics (ROC) curves were generated to determine the area under the curve (AUC) to evaluate the diagnostic ability of the ratios as prognostic markers. Youden’s score was used to detect optimal sensitivity and specificity.

As required, data are presented as absolute numbers with median, first and third quartile or as relative frequencies. SPSS^®^ Statistics version 27 (IBM, Armonk, NY, USA) was used and results with *p* < 0.05 were considered statistically significant.

## Results

161 pregnancies affected by FGR and 172 SGA pregnancies were included in our study. Of all eligible cases, 4 cases of stillbirth, 2 neonatal deaths and 6 terminations of pregnancy were excluded, leaving 333 patients for final analysis.

Maternal sociodemographic and obstetric characteristics as well as intrapartum outcomes are presented in Table 1. The median time interval between the last prenatal ultrasound and collection of data at birth was 1.29 (0.50–4.57) weeks.

**Table 1:** Maternal demographics, ultrasound characteristics and intrapartum outcomes stratified by FGR and SGA

Variables	FGR *n* = 161 (48.3%)	SGA *n* = 172 (51.7%)	*p*
Age of mother (years) median	30 [27–34]	30 [26–34]	0.656
GA_scan (weeks) median	32.8 [29.8–35.4]	34.9 [31.5–37.1]	< 0.001
Diabetes (maternal)	14 (8.7%)	5 (2.9%)	0.023
Smoker	7 (4.3%)	8 (4.7%)	0.894
Hypertension, pre-eclampsia (maternal)	10 (6.2%)	7 (4.1%)	0.376
UA PI median	1.21 [1.02–1.52]	1.01 [0.89–1.14]	< 0.001
MCA PI median	1.46 [1.26–1.79]	1.67 [1.40–1.94]	0.001
UCR median	0.86 [0.60–1.23]	0.62 [0.53–0.74]	< 0.001
CPR median	1.17 [0.82–1.66]	1.62 [1.35–1.90]	< 0.001
MCA PSV median	50.5 [42.5–56.9]	53.0 [45.3–59.3]	0.08
GA at delivery (weeks) median	34.8 [31.5–37.4]	38.1 [37.1–39.5]	< 0.001
APGAR after 5 min median	9 [8–10]	9 [9–10]	< 0.001
Birth weight centile			
< 3. Centile	70 (43.5%)	60 (34.9%)	0.109
< 10. Centile	112 (69.6%)	111 (64.5%)	0.330
Umbilical cord pH median	7.31 [7,27–7,34]	7.29 [7,25–7,34]	0.08
Birth weight (g) median	1780 [1230–2290]	2565 [2278–2805]	< 0.001
Mode of delivery			< 0.001
Spontaneous vaginal	29 (18.0%)	87 (50.6%)	
Instrumental	4 (2.5%)	7 (4.1%)	
Cesarean section	128 (79.5%)	78 (45.4%)	
CS primary Misgav-Ladach-Sectio	107 (66.5%)	50 (29.1%)	
CS secondary Misgav-Ladach-Sectio	21 (13.0%)	28 (16.3%)	
Premature birth (< 37 week)	106 (65.8%)	36 (20.9%)	< 0.001

Data are given as mean (SD); median (25th–75th centile) or number (%); data available for 333 women

*GA* gestational age, *GA_scan* gestational age at time of ultrasound scan, *UA* umbilical artery, *MCA* middle cerebral artery, *CPR* cerebroplacental ratio, *UCR* umbilicocerebral ratio, *PI* pulsatily index, *PSV* peak systolic velocity, *CS* Cesarean section

The median gestational age at time of ultrasound scan (GA_scan) differed between groups, with the FGR group showing an earlier GA_scan than the SGA group (32.8 [29.8–35.4] vs. 34.9 [31.5, 37.1] weeks; *p* < 0.001). Maternal age did not noticeably differ between groups. In the FGR cohort, the prevalence of maternal diabetes was higher (8.7% vs. 2.9%; *p* = 0.023), while no differences were noted in regard to hypertensive disorders or smoking (4.3% vs. 4.7%, *p* = 0.894; 6.2% vs. 4.1%; *p* = 0.376).

Median GA at delivery was noticeably smaller in the FGR cohort (34.8 [31.5, 37.4] weeks vs. 38.1 [37.1, 39.5] weeks; *p* < 0.001), the incidence of premature birth was over three times as high as in the SGA group (65.8% vs. 20.9%; *p* < 0.001).

Median birth weight in FGR pregnancies was lower (1780 g [1230, 2290] vs. 2565 g [2278, 2805]; *p* < 0.001) which is coherent with the lower median GA at delivery in the FGR group. There were equal cases with birth weight below the 10th centile (69.6% vs. 64.5%; *p* = 0.330) and more cases with birth weight centiles below 3 (43.5% vs. 34.9%; *p* = 0.109) centile in this group compared to the SGA cohort, although statistical significance was not reached.

There was a noticeably higher incidence of CS (79.5% vs. 45.4%; *p* < 0.001) and fewer spontaneous vaginal deliveries in the FGR group than in the SGA group (18.0% vs. 50.6%; *p* < 0.001).

There was no difference between the groups concerning the incidence of instrumental deliveries or median umbilical cord pH.

**Fig. 1:**
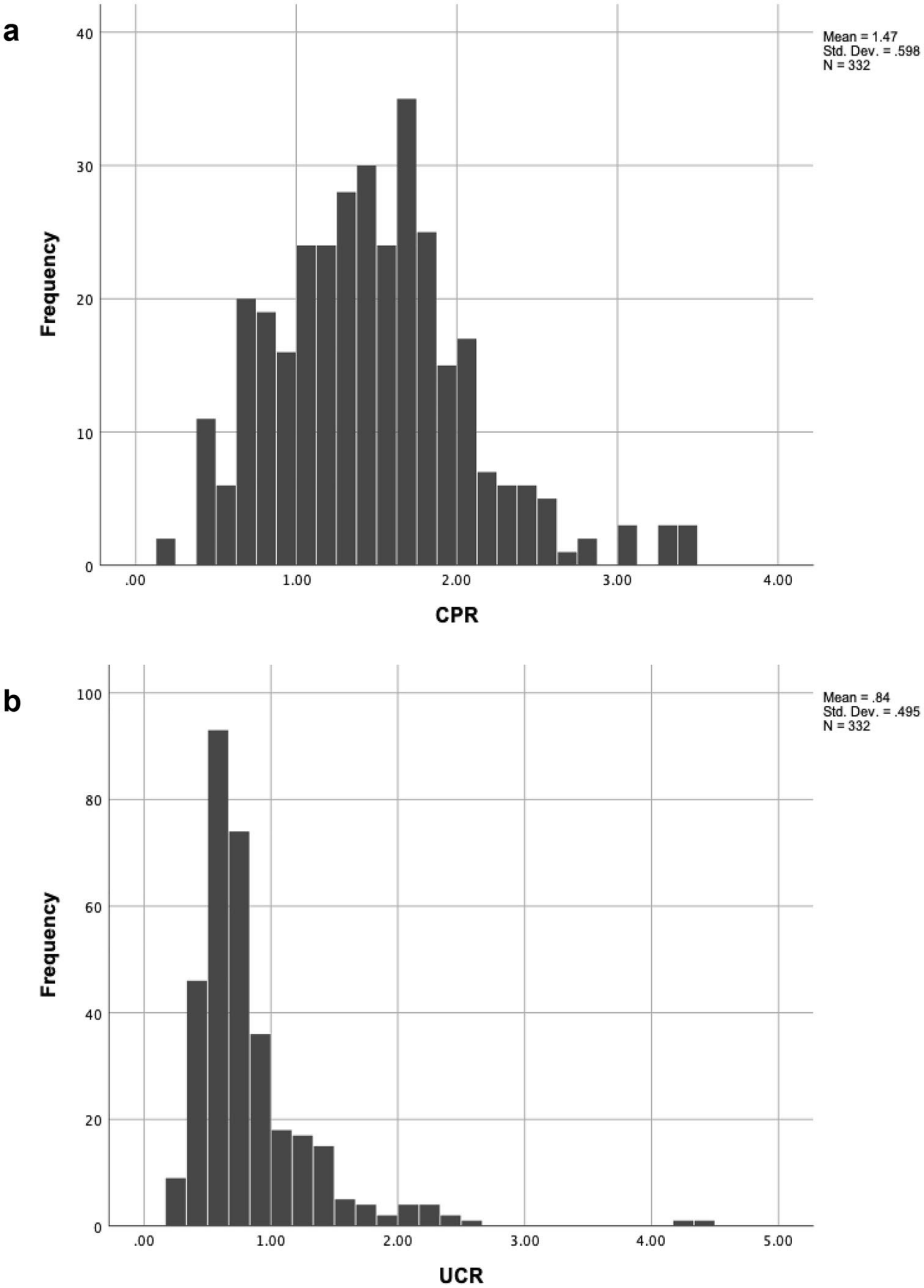
Distribution of CPR and UCR. **a** CPR shows a more symmetric distribution, **b** UCR′s distribution is asymmetrical with a right skew

Pregnancies affected by FGR presented a lower median CPR and conversely higher median UCR (1.17 vs. 1.62; *p* < 0.001; 0.86 vs. 0.62; *p* < 0.001, respectively) which can be traced back to FGR being defined by abnormal Doppler parameters. Neither ratio showed a normal distribution within either group according to the Kolmogorov–Smirnov and Shapiro–Wilk-tests. When comparing the distributions of CPR and UCR, CPR tended towards a more symmetric distribution (Fig. 1a), while the values of UCR were asymmetrical with a skew to the right (Fig. 1b).

Boxplots of UCR and CPR show the distribution of the ratios (SI 1): UCR shows a more distinctive discrimination of abnormal values (> 1) with outliers becoming more apparent.

In binary logistic regression analysis (Table 2), UCR showed independent association with preterm delivery under 37 and 30 weeks of gestation (OR: 5.857, 2.235–15.347; *p* = 0.001; OR: 2.908, 1.468–5.761; *p* = 0.001), APGAR score < 7 (OR: 3.529, 1.587–7.851; *p* = 0.001) and birth weight under the 10th centile (OR: 2.047, 0.979–4.280; *p* = 0.035) in pregnancies with FGR. CPR only showed an association with preterm delivery below 37 and 30 weeks (OR: 0.386, 0.224–0.666; *p* = 0.001; OR: 0.293, 0.119–0.721; *p* = 0.002) and APGAR score below 7 (OR: 0.273, 0.066–1.134; *p* = 0.04). In almost all parameters examined, UCR presented lower *p* values than CPR.

**Table 2:** Results of binary logistic regression analysis for FGR pregnancies

	Odds ratio (CI 95%)		*p* value^1^	
	CPR	UCR	CPR	UCR
Premature birth < 37	0.386 (0.224–0.666)	5.857 (2.235–15.347)	< 0.001^1^	< 0.001^1^
Premature birth < 30	0.293 (0.119–0.721)	2.908 (1.468–5.761)	0.002^1^	0.001^1^
APGAR < 7	0.273 (0.066–1.134)	3.529 (1.587–7.851)	0.040^1^	0.001^1^
Acidosis (pH < 7.2)	1.142 (0.419–3.114)	1.604 (0.681–3.782)	0.799	0.327
Weight < 10th centile	0.654 (0.401–1.064)	2.047 (0.979–4.280)	0.086	0.035^1^
Cesarean section	0.571 (0.336–0.972)	2.964 (1.091–8.051)	0.042^1^	0.014^1^

*CPR* cerebroplacental ratio, *UCR* umbilicocerebral ratio, *CI* confidence interval

^1^Significant as *p* < 0.05

Similar results were found when combining UCR or CPR with multiple ultrasound parameters in order to differentiate between SGA and FGR during pregnancy: odds ratios for UCR were highly significant compared to the odds ratios for CPR (OR: 0.065, 0.168–0.901; *p* = 0.027; OR: 0.810, 0.369–1.781; *p* = 0.601) as can be seen in Table 3.

**Table 3:** Logistic regression analysis for determination of FGR vs. SGA by combining CPR and UCR with different ultrasound parameters

	Odds ratio (CI 95%)	*p* value
UCR	0.065 (0.168–0.901)	0.027^1^
UA PI	0.065 (0.019–0.223)	< 0.001^1^
MCA PI	1.194 (0.592–2.405)	0.620
MCA PSV	0.993 (0.966–1.021)	0.620
GA_scan	1.053 (0.961–1.154)	0.268
CPR	0.810 (0.369–1.781)	0.601
UA PI	0.031 (0.007–0.142)	< 0.001^1^
MCA PI	1.991 (0.783–5.062)	0.148
MCA PSV	0.992 (0.965–1.019)	0.547
GA_scan	1.065 (0.973–1.167)	0.171

*CPR* cerebroplacental ratio, *UCR* umbilicocerebral ratio, *PI* pulsatility index, *UA* umbilical artery, *MCA* middle cerebral artery, *PSV* peak systolic velocity, *GA_scan* gestational age at time of ultrasound scan

^1^Significant as < 0.05

**Fig. 2:**
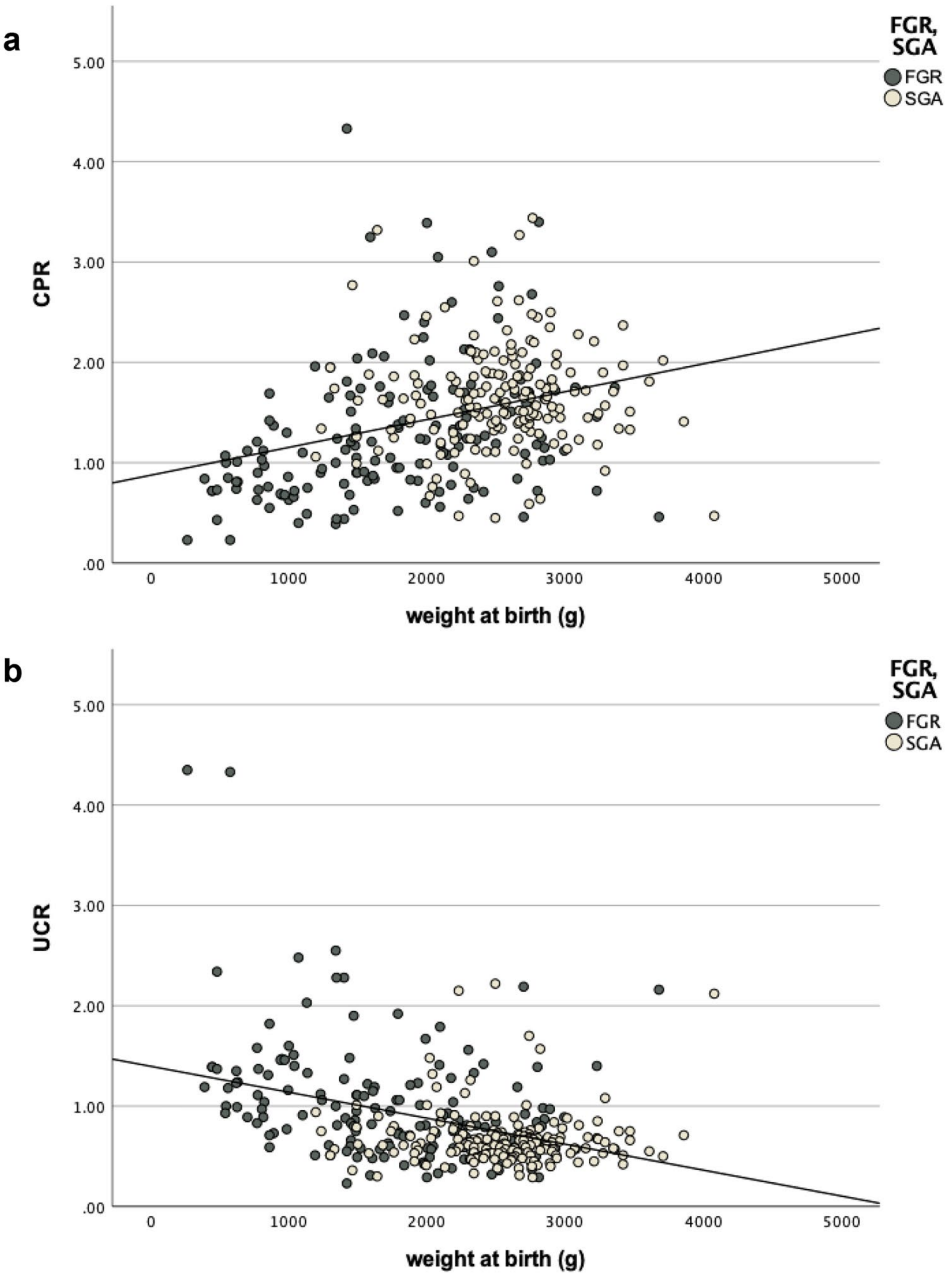
Scatter Plot of UCR and CPR by birth weight (g) separated by FGR and SGA. **a** CPR presents a moderate non-linear relationship with weight at birth, **b** UCR shows a stronger linear relationship with birth weight

Scatterplots for UCR and CPR with adverse outcome parameters identified different correlational relationships between the variables: UCR showed linear correlations with different negative outcome parameters, such as birth weight (Fig. 2a) and GA_birth, while the scatterplots for CPR illustrated more non-linear relationships (Fig. 2b).

Using different outcome parameters ROC plotted for CPR and UCR showed almost identical moderate prediction performance regarding *p* values and specificity as well as AUC. Highest predictive accuracies of CPR and UCR were found for premature birth < 37 weeks (AUC-CPR: 0.701 vs. 0.702; Sens: 0.557 vs. 0.604, Spec: 0.800 vs. 0.875), < 30 weeks (AUC: 0.723 vs. 0.722; Sens: 0.828; Spec: 0.598) and for an APGAR score below 7 (AUC: 0.708; Sens: 0.727, Spec: 0.693). Neonatal acidosis performed poorest with an AUC of 0.461. Moderate results were found for the prediction of birth weight below the 10th and 3rd centile, CS and instrumental delivery. Different cut-off values for CPR and UCR are shown in Table 4.

**Table 4:** Receiver operating characteristic curve analysis: AUC, CPR and UCR screening efficacy for adverse outcome parameters

Outcome	CPR				UCR			
	AUC	Sens	Spec	Cut-off	AUC	Sens	Spec	Cut-off
Premature birth < 37	0.701	0.557	0.8	1.075	0.702	0.604	0.745	0.875
Premature birth < 30	0.723	0.828	0.598	1.125	0.722	0.828	0.598	0.885
APGAR < 7	0.708	0.727	0.693	0.925	0.708	0.727	0.693	1.08
Acidosis < 7.2	0.461	0.25	0.889	0.67	0.460	0.25	0.889	1.495
centile < 10	0.597	0.518	0.706	1.095	0.597	0.518	0.706	0.915
centile < 3	0.538	0.456	0.667	1.005	0.538	0.456	0.667	0.995
MOD (obstetric intervention)	0.632	0.538	0.724	1.155	0.632	0.538	0.724	0.865
CS	0.651	0.547	0.727	1.155	0.651	0.547	0.727	0.865

*CPR* cerebroplacental ratio, *UCR* umbilical-to-cerebral ratio, *MOD* mode of delivery, *CS* Cesarean section

When combining different ultrasound parameters in forward stepwise regression analysis for the prediction of adverse outcome parameters (Table 5) a combination of GA_scan and UA PI showed highest predictive performance for the parameters premature birth, APGAR score below 7, and weight below the 10th and 3rd centile. For the prediction of premature birth (< 30th week) and birth weight below 3rd centile adding MCA PSV to UA PI and GA_scan increased predictive accuracy. The only parameter suitable for the prediction of CS was UA PI (OR: 14.765, 5.251–41.516; *p* < 0.001).

**Table 5:** Multivariate logistic regression analysis for prediction of adverse outcome by combining different parameters

	Parameters^1^	*p* value^2^	OR (CI 95%)
Premature birth < 37	GA_scan	0.000	41.195 (11.89–142.72)
	UA PI	0.000	0.824 (0.763–0.891)
Premature birth < 30	MCA PSV	0.003	1.142 (1.047–1.244)
	UA PI	0.00	21.237 (4.354–103.6)
	GA_scan	0.00	0.278 (0.165–0.468)
Cesarean section	UA PI	0.000	14.765 (5.251–41.516)
APGAR < 7	UA PI	0.038	2.21 (1.046–4.667)
	GA_scan	0.012	0.838 (0.730–0.962)
Weight < 10th centile	UA PI	0.001	4.6 (1.904–11.113)
	GA_scan	0.000	1.156 (1.081–1.236)
Weight < 3rd centile	MCA PSV	0.011	0.973 (0.954–0.994)
	UA PI	0.041	2.313 (1.036–5.166)
	UCR	0.003	0.325 (0.153–0.688)

^1^Parameters that showed highest predictive values for adverse outcome in forward stepwise analysis

^2^Significant as < 0.05

## Discussion

The results of our study demonstrated that while UCR and CPR reach similar prognostic accuracy concerning overall outcome, using UCR as a model shows better correlations with negative outcome parameters. In our analysis, UCR showed a higher association with outcome parameters as well as more noticeable *p* values for most tests performed. When graphically visualizing both ratios, the presentation of UCR confirmed its ability to better model an association with high-risk pregnancies.

In recent years, focus in prenatal diagnostic was set on establishing CPR as standard prediction marker for assessing adverse perinatal outcome to determine optimal timing of birth, while little attention was given to the prognostic relevance of UCR.

Our main test results for the predictive accuracy of CPR were consistent with the values previously published: CPR was associated with adverse pregnancy outcomes including preterm delivery and APGAR score below 7 [2, 8, 11, 16, 17], but showed poor results in the prediction of a low umbilical cord pH [10, 14]. Our ROC analysis showed similar AUC results for adverse neonatal outcome, our cut-off values (< 1.076) were similar to the values published in recent literature (< 1.08) [18, 19]. In a direct comparison of CPR and UCR, the statistical analysis of our study demonstrated that both ratios were equally associated with various outcome parameters and reached similar results regarding sensitivity and specificity in ROC analysis. However, using the model of UCR as a prognostic marker seemed to be more compatible in the context of predicting an adverse neonatal outcome.

This is in accordance with a recent study that analyzed whether establishing UCR instead of CPR adds any benefit to the prediction of adverse outcomes in singleton pregnancies. While similar AUC values were generated, the study of Leavitt et al. was limited to the direct comparison between logistic regression analysis and ROC curve results without including any additional Doppler parameters or the effects of the varying distribution of the models CPR and UCR [20].

The different performances of CPR and UCR were previously described by a secondary analysis of the TRUFFLE study [12] for the assessment of neonatal and 2-year infant outcome in early fetal growth restricted pregnancies. Stampalija et al. outlined how the odds ratio associated with UCR z-scores presented better associations with outcome parameters, while CPR z-scores showed no association with neonatal outcome. Similar observations were made by Familiari et al. in a study for a different high-risk collective with pregnancies affected by gestational diabetes [14].

Possible explanations for the differences in the results of statistical tests can be found in the different distribution of the ratios leading to converse behavior in abnormal range: while the values of the CPR are compressed trending towards zero, the UCR strives towards an asymptote leading towards infinity. Consistent with our own findings, it becomes evident that with increased alteration of fetal Doppler indices the effect on the UCR grows exponentially, allowing it to distinguish the collective with a negative outcome (SI 1). Abnormal outliers become more apparent and differentiate the extent of abnormality more clearly. This may also have an impact on the different correlational relationships of UCR and CPR with numeric variables as illustrated in the scatterplots in Fig. 2. UCR shows a better linear correlation with parameters measured to determine a negative outcome, which makes it a better fit for prognostic assessment leading to lower p values in statistical tests (Tables 2, 3).

This ultimately raises the question whether the resulting statistical discrepancy can be traced back to this simple mathematical relation of inversing the ratios of ACM PI and UA PI. It is necessary to reflect whether the noted statistical differences may result from wrong application or interpretation of statistical models: inversing the ratio leads to a change in the distribution, as can be seen in Fig. 1, which may lead to challenges in the further analysis of data. Kalafat et al. pointed out that the inaccuracies provoked using statistical tests that make assumptions about data distribution lead to biased results [21].

In accordance with a previous study that deals with a low-risk collective, we believe neither ratio to be adequate as sole screening marker, but that CPR and UCR only gain clinical relevance when combined with other parameters under specific conditions indicating a high-risk collective such as FGR—where minimization of very poor outcomes without significantly increasing the rate of cesarean sections and admissions to NICU should be the primary objective. It is important to consider these limitations when implementing CPR and UCR into clinical practice [22].

Our research showed good results when PI UA and GA_scan are combined for the prediction of adverse neonatal outcomes. We therefore propose establishing these two parameters as essential standard prediction markers for pregnancies at risk and to add other parameters such as UCR or CPR for further diagnosis.

One major limitation of previous studies is the inconsistent or even synonymous use of SGA and FGR, impeding direct comparison of results [12, 23–25]. As our study demonstrates a discrepancy regarding the outcomes of SGA pregnancies compared to FGR pregnancies (Table 1), we propose a consistent standardization of terminology and a universal consensus of defining FGR: SGA should refer to fetuses with smallness (weight < 10th centile), while FGR should be used for small fetuses with underlying pathologies such as abnormal Doppler indices or oligohydramnios [6].

To assess the predictive value of CPR regarding fetal outcome, Gramellini et al., among other researchers, used different categorical cut-off values ranging from < 1.0 to < 1.1 [18, 19, 26] for CPR while others established gestational age-specific normograms based on cross-sectional [27, 28] or longitudinal studies [29]. Odibo et al. found similar efficiency and prognostic utility of CPR cut-off values compared to the use of age-based thresholds, making neither method superior [8].

To the best of our knowledge, there are longitudinal reference ranges [13] as well as reference charts with different thresholds for CPR and UCR concerning adverse perinatal outcome [30]. Our average cut-off point for UCR was 0.925 (values ranging from 0.865–1.08) which corresponds with the recently published adjusted odds ratios for UCR > 0.9 (> 1.75 MoM) and we agree that absolute cut-off values are more viable for clinical use. Our cut-off values for the prediction of different outcome parameters resulting from our ROC curve analysis can be seen in Table 5.

Customized centiles with ethnicity- and gender-specific norms for assessing perinatal risk are a promising approach to improving the detection of FGR and SGA—further research with our data is conceivable [31].

### Study limitations and strengths

Study limitations lie within the nature of the retrospective design. In retrospect, inter- and intra-researcher variability is unknown and reliability of correct and systematic measurements cannot be confirmed. During the study period, two different data bases were used to collect the data. Merging the two sets of data may have provoked systematic or technical biases which we were unaware of as well as incomplete or missing maternal data. Possible residual confounders by unmeasured factors may remain. Another potential limitation is the rather long time interval between ultrasound and delivery that might cause a loss of predictive validity.

One major strength of our study was the high number of cases that we identified using strict inclusion criteria. Unlike most other studies we used SGA fetuses instead of normal pregnancies as control collective. This allowed us to distinguish more precisely between growth-restricted pregnancies at risk of decompensation and pregnancies where no intervention is indicated.

Another advantage is the uniform high data quality, because all ultrasound examinations were performed by qualified investigators with adequate experience and high-end equipment.

## Conclusion

At present, no other effective intervention for FGR pregnancies has been approved aside from delivery. Previously, a low CPR was used as a marker for alterations in cerebral or placental blood vessels to predict poor perinatal outcome in particular for late onset FGR. In our study, UCR showed a similar prognostic accuracy to CPR, but a closer correlation to adverse outcome parameters.

Adding UA PI and GA_scan to logistic regression analysis increased the prognostic accuracy regarding negative outcomes. These findings indicate that the UCR should be prospectively examined as a prognostic tool, while keeping the statistical characteristics and challenges of reversing the ratio in mind.

Our study emphasizes the need for standardization of medical terms such as FGR and SGA to develop generally valid management protocols for FGR pregnancies.

## Supplementary Information

Below is the link to the electronic supplementary material.

Supplementary SI 1 Boxplots of CPR and UCR (SGA vs. FGR) to visualize discrimination in abnormal range: UCR shows a more distinctive discrimination of abnormal values (> 0.93) with outliers becoming more apparent. Values below 1.08 were considered abnormal for CPR. (DOCX 24 KB)

## Data Availability

All data and materials support our claims and comply with field standards.

## Code availability

All software applications support our claims and comply with field standards.
