# Supplementary material for: Role of umbilicocerebral and cerebroplacental ratios in prediction of perinatal outcome in FGR pregnancies
Source: Arch Gynecol Obstet. 2021 Oct 2;305(6):1383–92. doi: 10.1007/s00404-021-06268-4 (PMC9166852; doi:10.1007/s00404-021-06268-4)

**Title**: Role of umbilicocerebral ratio in prediction of perinatal outcome in FGR pregnancies

**Journal**: Archives of Gynecology and Obstetrics

**Author name:** Hannah Coenen

**Affiliation:** Department of Gynecology and Obstetrics, University Hospital Münster, Albert-Schweitzer-Campus 1, 48149 Münster, Germany

**E-mail:** h_coen01@uni-muenster.de

**SI 1** Boxplots of CPR and UCR (SGA vs. FGR) to visualize discrimination in abnormal range


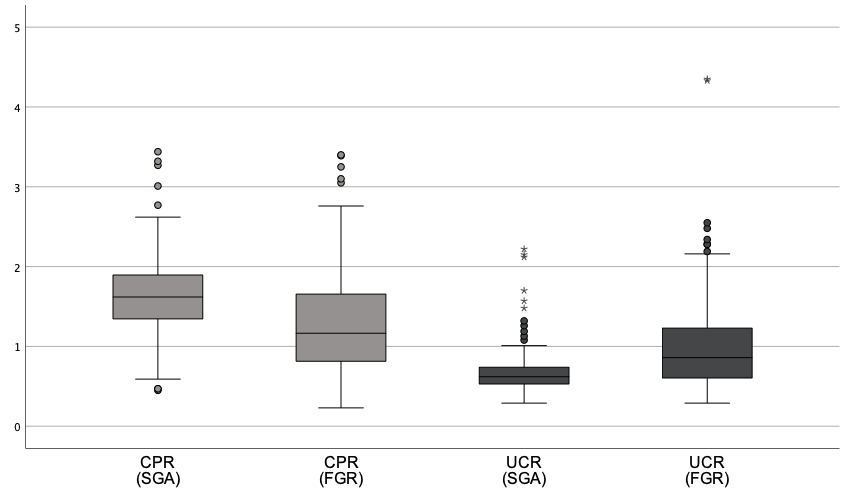

Supplement: Supplementary file 1 — Supplementary SI 1 Boxplots of CPR and UCR (SGA vs. FGR) to visualize discrimination in abnormal range: UCR shows a more distinctive discrimination of abnormal values (> 0.93) with outliers becoming more apparent. Values below 1.08 were considered abnormal for CPR. (DOCX 24 KB) [file 404_2021_6268_MOESM1_ESM.docx]
